# Linking serum neurofilament light chain to arthritis: Insights from a national population-based study

**DOI:** 10.1097/MD.0000000000043098

**Published:** 2025-07-25

**Authors:** Mingjiang Liu, Kun Xia

**Affiliations:** a Hunan Key Laboratory of Medical Genetics of the School of Life Sciences, Central South University, Changsha, China; b MOE Key Laboratory of Rare Pediatric Diseases, Hengyang Medical School, University of South China, Hengyang, China.

**Keywords:** arthritis, biomarker, inflammation, NHANES, Serum neurofilament light chain

## Abstract

Arthritis affects millions globally and imposes significant burdens on individual well-being and public health systems. Serum neurofilament light chain (sNFL) has been implicated in various neuroinflammatory and systemic conditions. However, its potential association with arthritis has not been explored in population-based settings. We analyzed data from the 2013 to 2014 National Health and Nutrition Examination Survey, including 2068 adults aged 20 years or older with available sNFL measurements and arthritis status based on self-reported physician diagnosis. Serum sNFL concentrations were quantified using a high-sensitivity chemiluminescence-based immunoassay. Multivariable logistic regression was used to estimate the association between sNFL levels and the odds of arthritis, adjusting for sociodemographic, lifestyle, and clinical factors. Subgroup and interaction analyses were performed to examine effect modification. Elevated serum sNFL levels were significantly associated with higher odds of having arthritis. After full covariate adjustment, each 1-unit increase in log-transformed sNFL was associated with an 85% increase in arthritis odds (odd ratio = 1.85; 95% confidence interval: 1.79–2.39). Participants in the highest sNFL quartile had 3.67 times higher odds of arthritis compared to those in the lowest quartile (*P* for trend = .003). Subgroup analyses indicated stronger associations in individuals with higher education and those without diabetes or hypertension (*P* for interaction < .05). This nationally representative analysis demonstrates a significant association between circulating sNFL levels and the presence of arthritis, suggesting that sNFL may reflect broader systemic or inflammatory processes linked to joint pathology. These findings support further investigation of sNFL as a potential biomarker for arthritis risk stratification.

## 1. Introduction

Arthritis, a collective term for over 100 joint-related disorders including osteoarthritis, rheumatoid arthritis, and psoriatic arthritis, represents one of the most prevalent chronic conditions worldwide.^[1]^ It significantly impairs physical function and quality of life, particularly among older adults.^[2,3]^ In the United States alone, approximately 1 in 4 adults report a physician diagnosis of arthritis, underscoring its substantial public health burden.^[4]^ Despite the widespread prevalence, early detection and risk stratification remain suboptimal, partly due to a lack of reliable blood-based biomarkers that capture underlying pathophysiological changes.

Serum neurofilament light chain (sNFL) is a cytoskeletal protein released into the bloodstream following axonal injury and has emerged as a sensitive marker of neurodegeneration.^[5,6]^ Its utility has been extensively validated in neurological conditions such as multiple sclerosis, Parkinson disease, and Alzheimer disease, where sNFL levels correlate with disease activity and prognosis.^[7,8]^ Beyond neurodegenerative diseases, recent evidence suggests that sNFL is also elevated in systemic inflammatory conditions including periodontitis and is associated with increased risk of mortality.^[9–11]^

Given the known interplay between systemic inflammation, neuro-immune regulation, and joint pathology, sNFL may also reflect processes relevant to arthritis. However, no prior population-based studies have assessed the association between circulating sNFL levels and arthritis prevalence. The current study aims to fill this gap by investigating the relationship between serum sNFL concentrations and self-reported arthritis in a nationally representative sample of U.S. adults, using data from the National Health and Nutrition Examination Survey (NHANES) 2013 to 2014 cycle.

## 2. Methods

### 2.1. Study population

This cross-sectional analysis utilized publicly available data from the 2013 to 2014 NHANES, a continuous, multistage probability survey conducted by the U.S. National Center for Health Statistics to assess health and nutritional status of the civilian, noninstitutionalized population.^[12]^ The NHANES protocol was approved by the National Center for Health Statistics Research Ethics Review Board, and all participants provided written informed consent.

Participants were eligible for inclusion if they were aged 20 years or older and had available data on serum neurofilament light chain concentrations and self-reported arthritis status. We excluded individuals under 20 years (n = 4406), those with missing sNFL measurements (n = 3698), and those missing arthritis status (n = 3), resulting in a final analytic sample of 2068 participants.

### 2.2. Measurement of serum neurofilament light chain

sNFL concentrations were measured using a high-sensitivity immunoassay developed by Siemens Healthineers, employing acridinium ester-based chemiluminescence on an automated Attelica platform.^[13]^ Briefly, serum samples were incubated with AE-labeled antibodies specific to neurofilament light chain and paramagnetic capture particles. After washing, chemiluminescence was triggered and quantified, with results expressed in picograms per milliliter (pg/mL).

### 2.3. Assessment of arthritis

Arthritis status was determined via structured questionnaires. Participants were asked, “Has a doctor or other health professional ever told you that you had arthritis?” Affirmative responses were coded as having arthritis, regardless of subtype. Those who responded “No” were categorized as non-arthritis. Additional arthritis subtype data were collected but not used for stratification in the present analysis.

### 2.4. Covariates

Covariates were selected a priori based on prior literature linking them to either arthritis or sNFL levels.^[13–15]^ These included age, sex, race/ethnicity (non-Hispanic White, non-Hispanic Black, Mexican American, and other), body mass index (BMI), education level (<high school, high school, >high school), poverty-income ratio, smoking status (ever vs never), diabetes status, hypertension status, total cholesterol, and high-density lipoprotein cholesterol (HDL-C). Laboratory measures followed standardized NHANES protocols, and smoking was defined as having smoked ≥100 cigarettes in a lifetime.

### 2.5. Statistical analysis

Analyses accounted for the complex survey design using appropriate NHANES weights, strata, and primary sampling units. Missing data were imputed in using random forest interpolation via the “missForest” package (1.5.0) in R.^[16,17]^ Descriptive statistics of participant characteristics were stratified by quartiles of sNFL. Continuous variables were summarized as weighted means with standard deviations, and categorical variables as unweighted counts with weighted percentages. Between-group differences were assessed using ANOVA or chi-square tests as appropriate.

To evaluate the association between sNFL and arthritis, multivariable logistic regression models were constructed. sNFL levels were log-transformed to normalize their distribution. Model 1 was unadjusted; Model 2 adjusted for age, sex, and race/ethnicity; and Model 3 further adjusted for education, BMI, poverty-income ratio, smoking, HDL-C, total cholesterol, diabetes, and hypertension. sNFL quartiles were also used to evaluate dose–response associations, with *P* for trend calculated across categories.^[18]^ Subgroup analyses were performed by sex, age, education, diabetes, and hypertension, and interaction terms were tested to assess effect modification. All analyses were conducted using R software (version 4.4.1), and statistical significance was defined as a 2-sided *P*-value < .05.

## 3. Results

### 3.1. Participant characteristics

A total of 2068 adults were included in the analysis, with a weighted mean age of 46.9 ± 13.6 years. Among them, 47.8% were male, and 52.2% were female. Overall, 22.9% of participants reported a diagnosis of arthritis by a healthcare professional. The prevalence of arthritis increased across quartiles of serum sNFL levels, from 6.95% in the lowest quartile (<7.1 pg/mL) to 43.63% in the highest quartile (>15.1 pg/mL) (Table 1).

**Table 1:** Characteristics of selected participants.

Characteristics	Total	Serum neurofilament light chain				*P*-value
		Quartile 1	Quartile 2	Quartile 3	Quartile 4	
Age, mean (SD)	46.87 ± 15.35	35.43 ± 10.67	43.99 ± 13.25	51.47 ± 14.36	56.46 ± 14.01	<.001
Sex, (%)						<.001
Male	989 (47.82%)	210 (41.02%)	251 (48.27%)	255 (49.23%)	273 (52.70%)	
Female	1079 (52.18%)	302 (58.98%)	269 (51.73%)	263 (50.77%)	245 (47.30%)	
Race/ethnicity, n (%)						<.001
Non-Hispanic White	909 (43.96%)	191 (37.30%)	210 (40.38%)	249 (48.07%)	259 (50.00%)	
Non-Hispanic Black	372 (17.99%)	93 (18.16%)	110 (21.15%)	71 (13.71%)	98 (18.92%)	
Mexican American	292 (14.12%)	99 (19.34%)	74 (14.23%)	52 (10.04%)	67 (12.93%)	
Other races	495 (23.94%)	129 (25.20%)	126 (24.23%)	146 (28.19%)	94 (18.15%)	
Education level, n (%)						.199
<High school	449 (21.74%)	113 (22.11%)	106 (20.42%)	102 (19.69%)	128 (24.76%)	
High school	431 (20.87%)	95 (18.59%)	117 (22.54%)	105 (20.27%)	114 (22.05%)	
>High school	1185 (57.38%)	303 (59.30%)	296 (57.03%)	311 (60.04%)	275 (53.19%)	
Diabetes, n (%)						<.001
No	1783 (86.22%)	486 (94.92%)	480 (92.31%)	434 (83.78%)	383 (73.94%)	
Yes	285 (13.78%)	26 (5.08%)	40 (7.69%)	84 (16.22%)	135 (26.06%)	
Hypertension, n (%)						<.001
No	1336 (64.60%)	415 (81.05%)	366 (70.38%)	309 (59.65%)	246 (47.49%)	
Yes	732 (35.40%)	97 (18.95%)	154 (29.62%)	209 (40.35%)	272 (52.51%)	
Arthritis, n (%)						<.001
No	1595 (77.13%)	481 (93.95%)	431 (83.88%)	391 (75.48%)	292 (56.37%)	
Yes	473 (22.87%)	31 (6.05%)	89 (16.12%)	127 (24.52%)	226 (43.63%)	
PIR, mean (SD)	29.24 ± 7.37	29.75 ± 7.43	28.79 ± 7.45	28.54 ± 6.78	29.88 ± 7.70	.002
BMI, kg/m^2^, mean (SD)	2.49 ± 1.67	2.40 ± 1.64	2.57 ± 1.69	2.58 ± 1.72	2.43 ± 1.62	.270
HDL-C, mg/dL, mean (SD)	53.56 ± 15.79	52.66 ± 14.42	54.41 ± 14.81	54.24 ± 16.26	52.91 ± 17.46	.049
TC, mg/dL, mean (SD)	189.29 ± 40.76	185.02 ± 41.70	188.70 ± 36.64	192.84 ± 41.42	190.58 ± 42.76	.018

Data are presented as weighted mean (SD) for continuous variables and unweighted frequencies (weighted percentages) for categorical variables.

BMI = body mass index, HDL-C = high-density lipoprotein cholesterol, PIR = the ratio of income to poverty, Q = quartile, TC = total cholesterol.

Participants with higher sNFL levels tended to be older, male, non-Hispanic White, and more likely to have comorbid hypertension, diabetes, and cardiovascular disease (*P* < .001 for all). They also had lower income, reduced HDL-C levels, and higher total cholesterol. BMI and education level did not significantly differ across sNFL quartiles.

### 3.2. Relationship between sNFL and arthritis

As presented in Table 2, higher sNFL concentrations were significantly associated with increased odds of arthritis across all analytic models. In unadjusted analyses, each 1-unit increase in natural log-transformed sNFL corresponded to a 125% elevation in the odds of having arthritis. After adjusting for age, sex, and race/ethnicity, the association remained robust, with a 101% increase in arthritis risk. In the fully adjusted model controlling for sociodemographic, lifestyle, and clinical variables, a 1-unit increment in log-sNFL was still linked to an 85% higher likelihood of arthritis (odd ratio = 1.85; 95% confidence interval: 1.79–2.39).

**Table 2:** Relationship between serum neurofilament light chain levels and arthritis.

Exposure	Model 1 [OR (95% CI)]	Model 2 [OR (95% CI)]	Model 3 [OR (95% CI)]
Ln-transformed sNFL (continuous)	2.25 (1.95, 2.55)	2.01 (1.71, 2.31)	1.85 (1.79, 2.39)
sNFL (quartile)			
Quartile 1	Reference	Reference	Reference
Quartile 2	2.62 (1.63, 3.61)	2.51 (1.52, 3.50)	2.25 (1.35, 3.33)
Quartile 3	3.25 (2.09, 4.37)	3.11 (1.95, 4.23)	3.01 (1.84, 4.06)
Quartile 4	4.52 (2.99, 6.10)	4.12 (2.49, 5.70)	3.67 (2.32, 4.89)
*P* for trend	<.001	.001	.003

Model 1: No covariates were adjusted. Model 2: Age, sex and race were adjusted. Model 3: Age, sex, race, body mass index, education level, high-density lipoprotein cholesterol, total cholesterol, ratio of family income to poverty, diabetes status, and hypertension status were adjusted.

CI = confidence interval, OR = odds ratio, Q = quartile, sNFL = serum neurofilament light chain.

**Figure 1.**
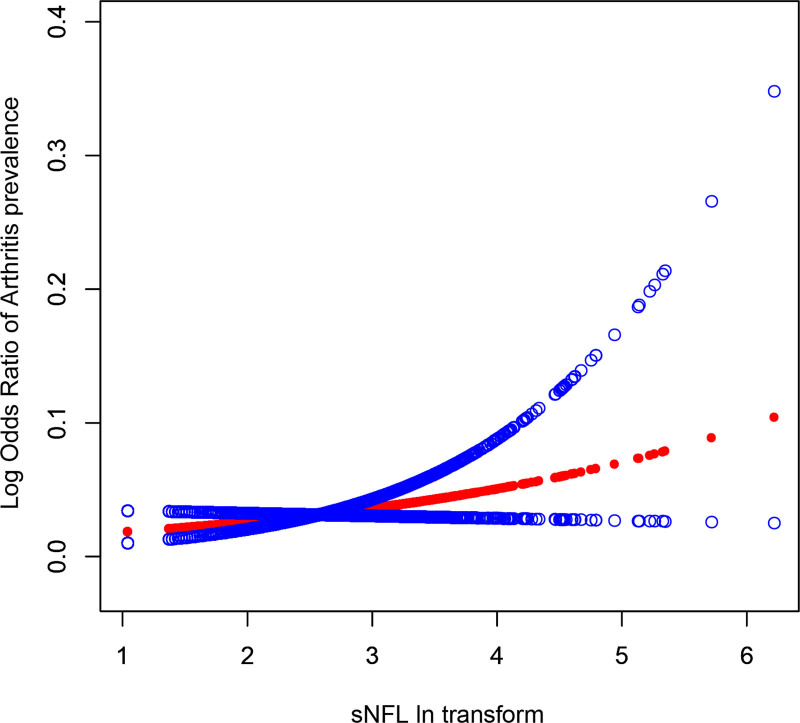
Dose–response relationship between serum neurofilament light chain (sNFL) levels and the prevalence of arthritis. The solid line represents the estimated odds ratio, and the shaded area denotes the 95% confidence interval. Models were adjusted for age, sex, race/ethnicity, body mass index, education level, high-density lipoprotein cholesterol, total cholesterol, poverty-income ratio, smoking status, diabetes, and hypertension.

Participants in the highest quartile of sNFL exhibited markedly greater odds of arthritis compared to those in the lowest quartile (odd ratio = 3.67; 95% confidence interval: 2.32–4.89), with a statistically significant dose–response trend (*P* for trend = .003). These findings were further supported by smoothed curve fitting analyses, which demonstrated a consistent positive relationship between serum sNFL levels and arthritis prevalence (Fig. 1).

**Figure 2.**
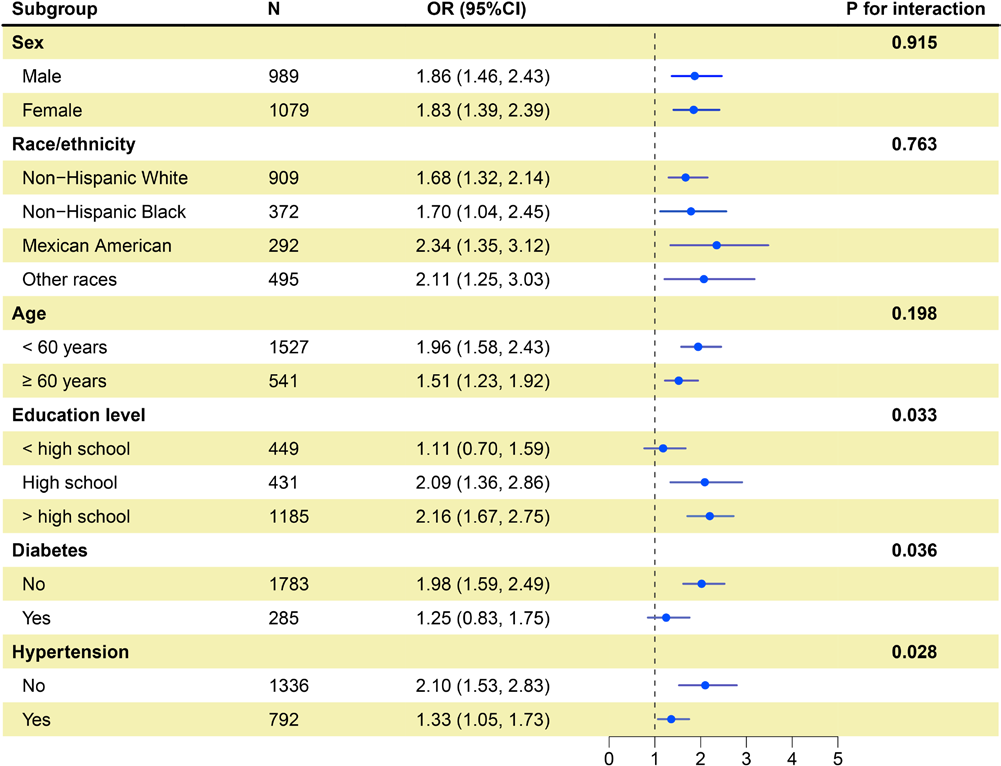
Subgroup analyses examining the association between sNFL levels and arthritis across different demographic and clinical categories. Models were adjusted for age, sex, race/ethnicity, body mass index, education level, high-density lipoprotein cholesterol, total cholesterol, poverty-income ratio, smoking status, diabetes, and hypertension. CI = confidence interval, OR = odds ratio, sNFL = serum neurofilament light chain.

Stratified analyses across subgroups are displayed in Figure 2. The positive association between sNFL and arthritis was generally observed regardless of sex, race/ethnicity, age group, educational level, hypertension, or diabetes status, although not all subgroup estimates achieved statistical significance. Interaction testing indicated that sex, race, and age did not significantly modify the sNFL-arthritis relationship (*P* for interaction > .05). However, educational attainment, hypertension status, and diabetes status significantly influenced the magnitude of association (*P* for interaction < .05). Specifically, the association between elevated sNFL levels and arthritis was stronger and statistically significant among individuals with higher educational levels, and among those without hypertension or diabetes (*P* < .05).

## 4. Discussion

In this nationally representative cohort of U.S. adults, we observed that higher sNFL levels were significantly associated with greater odds of arthritis, independent of sociodemographic, lifestyle, and cardiometabolic factors. To our knowledge, this is the first study to systematically examine sNFL in relation to arthritis as a comprehensive clinical outcome, encompassing osteoarthritis, rheumatoid arthritis, psoriatic arthritis, and other joint disorders.

Previous research has predominantly focused on the utility of sNFL as a sensitive biomarker for neuroaxonal injury in neurological conditions, including multiple sclerosis, Alzheimer disease, and Parkinson disease.^[8,19,20]^ Elevated sNFL concentrations have been shown to correlate with disease progression, disability accumulation, and cognitive decline in these contexts. More recently, sNFL has also been implicated in systemic inflammatory states and increased risk of all-cause mortality, suggesting its broader relevance beyond the nervous system.^[15,21]^ Our findings extend this body of evidence by demonstrating that sNFL may reflect systemic processes relevant to joint pathology as well.

The biological plausibility of our observations can be attributed to several overlapping mechanisms between neurodegeneration and arthritis. Chronic systemic inflammation, immune system dysregulation, oxidative stress, and endothelial dysfunction are hallmarks shared by both conditions.^[22–24]^ In arthritis, persistent synovial inflammation and subsequent structural joint damage are key pathophysiological processes, while in neurological diseases, similar inflammatory cascades contribute to blood–brain barrier breakdown and axonal loss.^[25]^ It is conceivable that elevated sNFL concentrations may serve as an integrated signal of inflammatory damage occurring concurrently in neural and joint tissues. Moreover, the role of oxidative stress in promoting cartilage degradation via matrix metalloproteinase activation parallels mechanisms seen in neuronal injury and warrants further exploration.^[26]^

Subgroup analyses revealed that the association between sNFL and arthritis was stronger among individuals without diabetes or hypertension. These findings suggest that cardiometabolic comorbidities, themselves linked to systemic inflammation and vascular compromise, might mask or attenuate the arthritis-specific relationship with sNFL. Diabetes and hypertension may independently elevate sNFL levels, potentially diluting the specific signal attributable to joint pathology alone.^[14,27]^ Future studies focusing on individuals without major metabolic disorders may help clarify the specificity of sNFL for arthritis risk stratification.

Interestingly, educational attainment appeared to modify the association, with a stronger relationship observed in participants with higher education levels. Several explanations could underlie this observation. Individuals with greater educational attainment may have better access to healthcare, greater health literacy, or earlier recognition and reporting of arthritis symptoms, leading to more accurate disease classification.^[28–30]^ Alternatively, lifestyle factors correlated with education, such as physical activity or occupational exposures, may also play a role.^[31,32]^ Nonetheless, the possibility of residual confounding cannot be entirely excluded.

From a clinical perspective, our findings suggest that serum sNFL measurement could potentially serve as a noninvasive, accessible biomarker to aid in the early identification of individuals at elevated risk for arthritis. Given the chronic and progressive nature of arthritis, early detection remains a critical unmet need. Incorporating biomarkers like sNFL into clinical risk models may enhance screening strategies, particularly for populations without overt clinical symptoms.

However, several limitations should be acknowledged when interpreting these results. First, the cross-sectional design of NHANES prevents determination of temporal or causal relationships between elevated sNFL and the onset of arthritis.^[33]^ Longitudinal studies are necessary to confirm whether increased sNFL levels precede clinical arthritis diagnosis. Second, arthritis status was based on self-report, which may be subject to misclassification bias, although prior studies have shown moderate agreement between self-report and physician diagnosis in large epidemiologic surveys. Third, despite extensive covariate adjustment, residual confounding by factors such as medication use, unmeasured comorbidities, or physical activity patterns remains possible. Finally, although sNFL reflects systemic injury, its specificity for arthritis as opposed to other inflammatory or degenerative conditions warrants further validation.

Despite these limitations, our study offers important novel insights. By leveraging a large, diverse, population-based cohort with rigorous biomarker measurement, we provide preliminary evidence supporting sNFL as a potential systemic biomarker associated with arthritis. These findings lay the groundwork for future prospective investigations aimed at validating sNFL’s role in arthritis risk prediction and disease monitoring.

## 5. Conclusion

In summary, this nationally representative study identifies a novel association between serum neurofilament light chain levels and the presence of arthritis, encompassing osteoarthritis, rheumatoid arthritis, psoriatic arthritis, and other joint disorders. Our findings suggest that sNFL, traditionally regarded as a marker of neuroaxonal injury, may also serve as a systemic biomarker reflecting joint-related pathological processes. Given the high global burden of arthritis and its profound impact on quality of life, the recognition of sNFL’s potential role in this context could inform future strategies for early detection, risk stratification, and disease monitoring. Further longitudinal research is warranted to establish temporal relationships and evaluate the clinical utility of sNFL in arthritis prevention and management.

## Author contributions

**Conceptualization:** Mingjiang Liu, Kun Xia.

**Data curation:** Mingjiang Liu.

**Formal analysis:** Mingjiang Liu.

**Funding acquisition:** Kun Xia.

**Investigation:** Mingjiang Liu.

**Methodology:** Mingjiang Liu.

**Project administration:** Mingjiang Liu, Kun Xia.

**Resources:** Mingjiang Liu.

**Software:** Mingjiang Liu.

**Supervision:** Kun Xia.

**Validation:** Mingjiang Liu.

**Visualization:** Mingjiang Liu.

**Writing – original draft:** Mingjiang Liu.

**Writing – review & editing:** Mingjiang Liu, Kun Xia.
